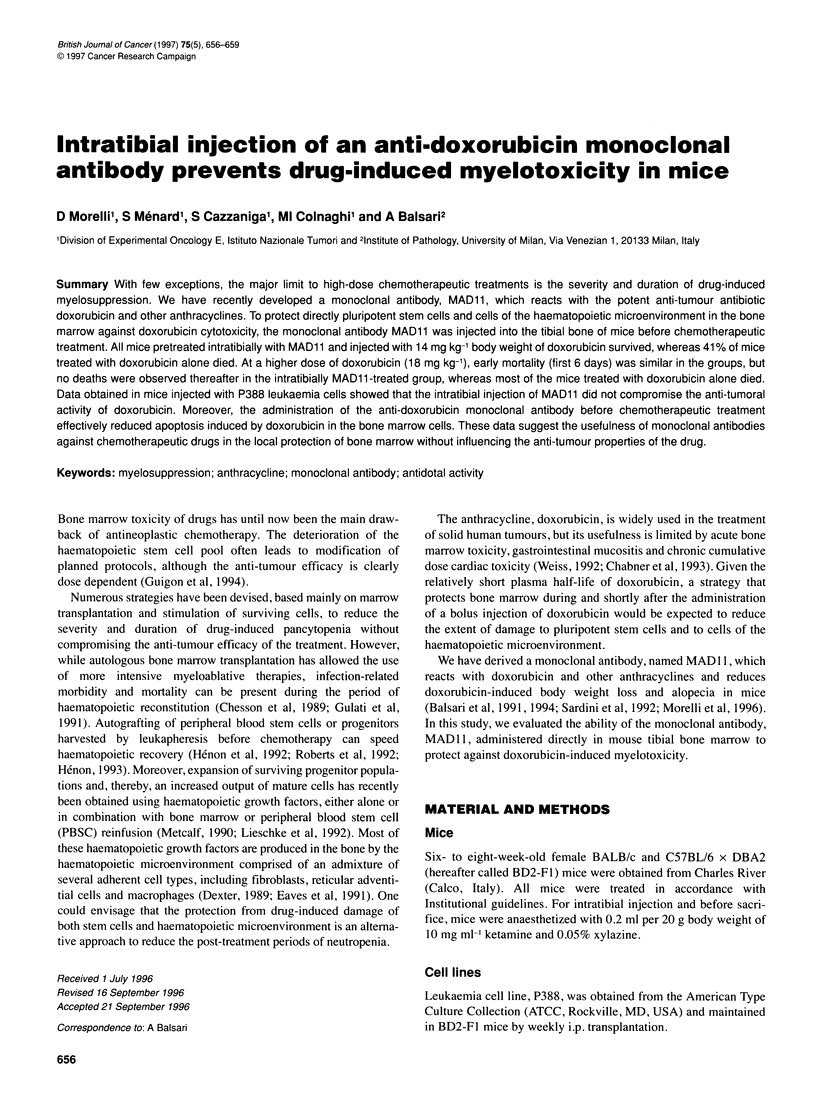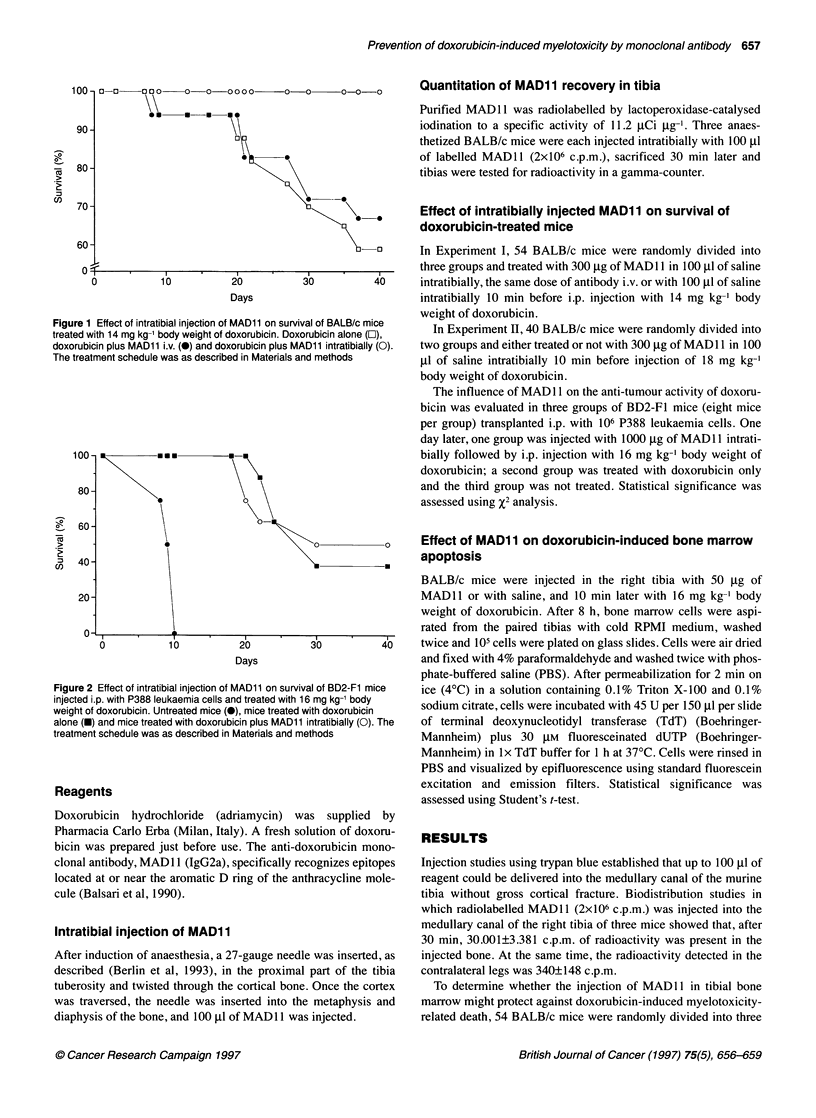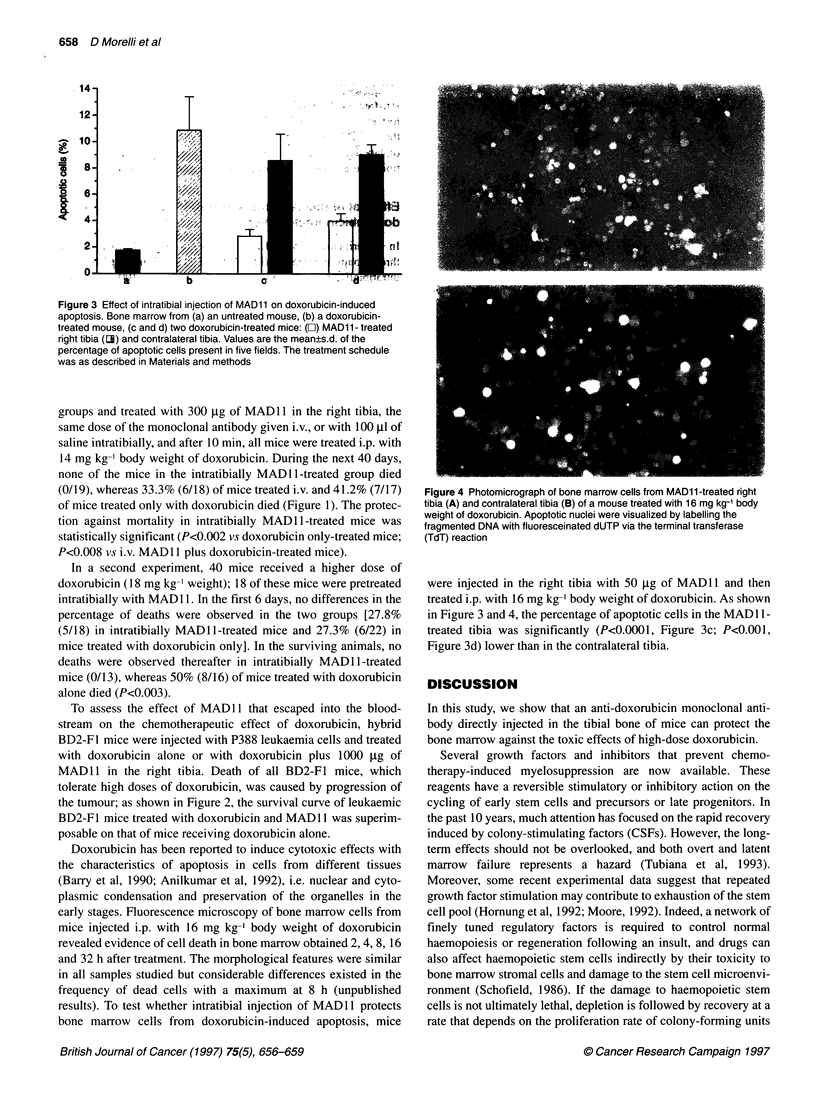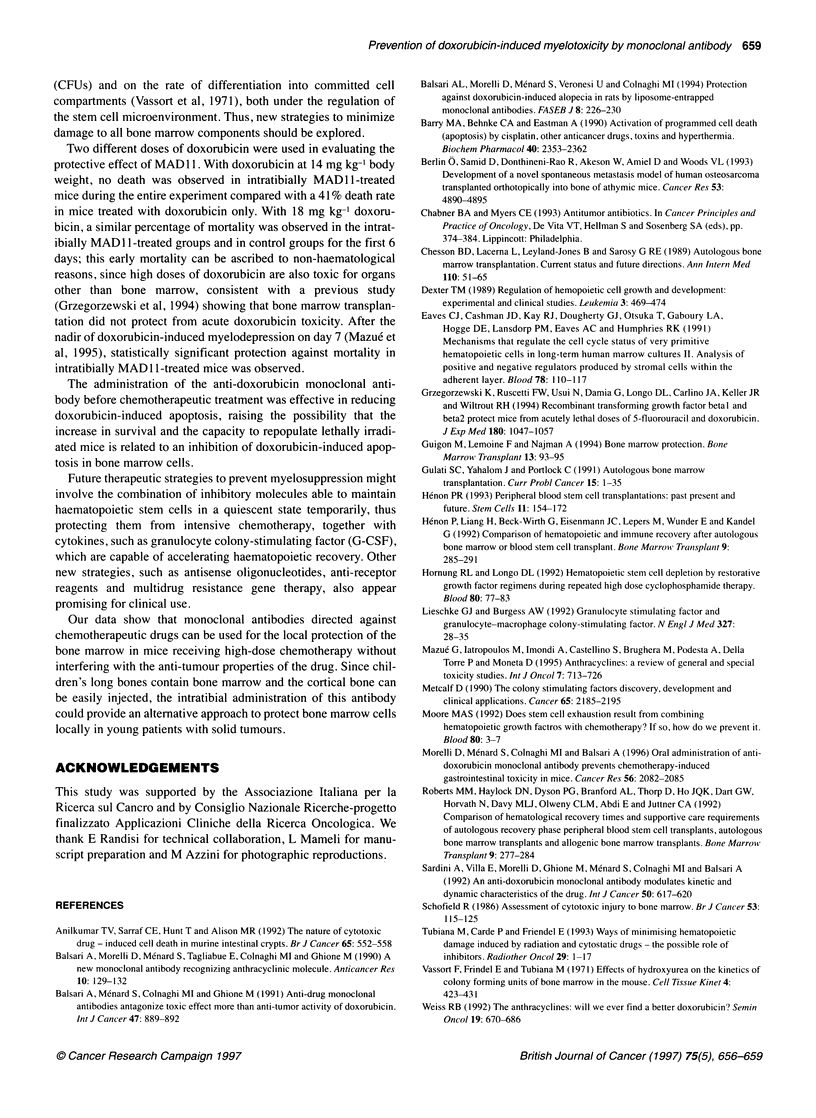# Intratibial injection of an anti-doxorubicin monoclonal antibody prevents drug-induced myelotoxicity in mice.

**DOI:** 10.1038/bjc.1997.117

**Published:** 1997

**Authors:** D. Morelli, S. Ménard, S. Cazzaniga, M. I. Colnaghi, A. Balsari

**Affiliations:** Division of Experimental Oncology E, Istituto Nazionale Tumori, University of Milan, Italy.

## Abstract

**Images:**


British Journal of Cancer (1997) 75(5), 656–659
© 1997 Cancer Research Campaign

### Intratibial injection of an anti-doxorubicin monoclonal antibody prevents drug-induced myelotoxicity in mice

**D Morelli[1], S Ménard[1], S Cazzaniga[1], MI Colnaghi[1] and A Balsari[2]**

[1]Division of Experimental Oncology E, Istituto Nazionale Tumori and [2]Institute of Pathology, University of Milan, Via Venezian 1, 20133 Milan, Italy

**Summary** With few exceptions, the major limit to high-dose chemotherapeutic treatments is the severity and duration of drug-induced myelosuppression. We have recently developed a monoclonal antibody, MAD11, which reacts with the potent anti-tumour antibiotic doxorubicin and other anthracyclines. To protect directly pluripotent stem cells and cells of the haematopoietic microenvironment in the bone marrow against doxorubicin cytotoxicity, the monoclonal antibody MAD11 was injected into the tibial bone of mice before chemotherapeutic treatment. All mice pretreated intratibially with MAD11 and injected with 14 mg $kg^{-1}$ body weight of doxorubicin survived, whereas 41% of mice treated with doxorubicin alone died. At a higher dose of doxorubicin (18 mg $kg^{-1}$), early mortality (first 6 days) was similar in the groups, but no deaths were observed thereafter in the intratibially MAD11-treated group, whereas most of the mice treated with doxorubicin alone died. Data obtained in mice injected with P388 leukaemia cells showed that the intratibial injection of MAD11 did not compromise the anti-tumoral activity of doxorubicin. Moreover, the administration of the anti-doxorubicin monoclonal antibody before chemotherapeutic treatment effectively reduced apoptosis induced by doxorubicin in the bone marrow cells. These data suggest the usefulness of monoclonal antibodies against chemotherapeutic drugs in the local protection of bone marrow without influencing the anti-tumour properties of the drug.

**Keywords:** myelosuppression; anthracycline; monoclonal antibody; antidotal activity

Bone marrow toxicity of drugs has until now been the main drawback of antineoplastic chemotherapy. The deterioration of the haematopoietic stem cell pool often leads to modification of planned protocols, although the anti-tumour efficacy is clearly dose dependent (Guigon et al, 1994).

Numerous strategies have been devised, based mainly on marrow transplantation and stimulation of surviving cells, to reduce the severity and duration of drug-induced pancytopenia without compromising the anti-tumour efficacy of the treatment. However, while autologous bone marrow transplantation has allowed the use of more intensive myeloablative therapies, infection-related morbidity and mortality can be present during the period of haematopoietic reconstitution (Chesson et al, 1989; Gulati et al, 1991). Autografting of peripheral blood stem cells or progenitors harvested by leukapheresis before chemotherapy can speed haematopoietic recovery (Hénon et al, 1992; Roberts et al, 1992; Hénon, 1993). Moreover, expansion of surviving progenitor populations and, thereby, an increased output of mature cells has recently been obtained using haematopoietic growth factors, either alone or in combination with bone marrow or peripheral blood stem cell (PBSC) reinfusion (Metcalf, 1990; Lieschke et al, 1992). Most of these haematopoietic growth factors are produced in the bone by the haematopoietic microenvironment comprised of an admixture of several adherent cell types, including fibroblasts, reticular adventitial cells and macrophages (Dexter, 1989; Eaves et al, 1991). One could envisage that the protection from drug-induced damage of both stem cells and haematopoietic microenvironment is an alternative approach to reduce the post-treatment periods of neutropenia.

*Received 1 July 1996*
*Revised 16 September 1996*
*Accepted 21 September 1996*

*Correspondence to*: A Balsari

The anthracycline, doxorubicin, is widely used in the treatment of solid human tumours, but its usefulness is limited by acute bone marrow toxicity, gastrointestinal mucositis and chronic cumulative dose cardiac toxicity (Weiss, 1992; Chabner et al, 1993). Given the relatively short plasma half-life of doxorubicin, a strategy that protects bone marrow during and shortly after the administration of a bolus injection of doxorubicin would be expected to reduce the extent of damage to pluripotent stem cells and to cells of the haematopoietic microenvironment.

We have derived a monoclonal antibody, named MAD11, which reacts with doxorubicin and other anthracyclines and reduces doxorubicin-induced body weight loss and alopecia in mice (Balsari et al, 1991, 1994; Sardini et al, 1992; Morelli et al, 1996). In this study, we evaluated the ability of the monoclonal antibody, MAD11, administered directly in mouse tibial bone marrow to protect against doxorubicin-induced myelotoxicity.

### MATERIAL AND METHODS

### Mice

Six- to eight-week-old female BALB/c and C57BL/6 × DBA2 (hereafter called BD2-F1) mice were obtained from Charles River (Calco, Italy). All mice were treated in accordance with Institutional guidelines. For intratibial injection and before sacrifice, mice were anaesthetized with 0.2 ml per 20 g body weight of 10 mg $ml^{-1}$ ketamine and 0.05% xylazine.

### Cell lines

Leukaemia cell line, P388, was obtained from the American Type Culture Collection (ATCC, Rockville, MD, USA) and maintained in BD2-F1 mice by weekly i.p. transplantation.

**Figure 1** Effect of intratibial injection of MAD11 on survival of BALB/c mice treated with 14 mg kg$^{-1}$ body weight of doxorubicin. Doxorubicin alone (□), doxorubicin plus MAD11 i.v. (●) and doxorubicin plus MAD11 intratibially (○). The treatment schedule was as described in Materials and methods

**Figure 2** Effect of intratibial injection of MAD11 on survival of BD2-F1 mice injected i.p. with P388 leukaemia cells and treated with 16 mg kg$^{-1}$ body weight of doxorubicin. Untreated mice (●), mice treated with doxorubicin alone (■) and mice treated with doxorubicin plus MAD11 intratibially (○). The treatment schedule was as described in Materials and methods

### Reagents

Doxorubicin hydrochloride (adriamycin) was supplied by Pharmacia Carlo Erba (Milan, Italy). A fresh solution of doxorubicin was prepared just before use. The anti-doxorubicin monoclonal antibody, MAD11 (IgG2a), specifically recognizes epitopes located at or near the aromatic D ring of the anthracycline molecule (Balsari et al, 1990).

### Intratibial injection of MAD11

After induction of anaesthesia, a 27-gauge needle was inserted, as described (Berlin et al, 1993), in the proximal part of the tibia tuberosity and twisted through the cortical bone. Once the cortex was traversed, the needle was inserted into the metaphysis and diaphysis of the bone, and 100 μl of MAD11 was injected.

### Quantitation of MAD11 recovery in tibia

Purified MAD11 was radiolabelled by lactoperoxidase-catalysed iodination to a specific activity of 11.2 μCi μg$^{-1}$. Three anaesthetized BALB/c mice were each injected intratibially with 100 μl of labelled MAD11 (2x10$^{6}$ c.p.m.), sacrificed 30 min later and tibias were tested for radioactivity in a gamma-counter.

### Effect of intratibially injected MAD11 on survival of doxorubicin-treated mice

In Experiment I, 54 BALB/c mice were randomly divided into three groups and treated with 300 μg of MAD11 in 100 μl of saline intratibially, the same dose of antibody i.v. or with 100 μl of saline intratibially 10 min before i.p. injection with 14 mg kg$^{-1}$ body weight of doxorubicin.

In Experiment II, 40 BALB/c mice were randomly divided into two groups and either treated or not with 300 μg of MAD11 in 100 μl of saline intratibially 10 min before injection of 18 mg kg$^{-1}$ body weight of doxorubicin.

The influence of MAD11 on the anti-tumour activity of doxorubicin was evaluated in three groups of BD2-F1 mice (eight mice per group) transplanted i.p. with 10$^{6}$ P388 leukaemia cells. One day later, one group was injected with 1000 μg of MAD11 intratibially followed by i.p. injection with 16 mg kg$^{-1}$ body weight of doxorubicin; a second group was treated with doxorubicin only and the third group was not treated. Statistical significance was assessed using $\chi^2$ analysis.

### Effect of MAD11 on doxorubicin-induced bone marrow apoptosis

BALB/c mice were injected in the right tibia with 50 μg of MAD11 or with saline, and 10 min later with 16 mg kg$^{-1}$ body weight of doxorubicin. After 8 h, bone marrow cells were aspirated from the paired tibias with cold RPMI medium, washed twice and 10$^{5}$ cells were plated on glass slides. Cells were air dried and fixed with 4% paraformaldehyde and washed twice with phosphate-buffered saline (PBS). After permeabilization for 2 min on ice (4°C) in a solution containing 0.1% Triton X-100 and 0.1% sodium citrate, cells were incubated with 45 U per 150 μl per slide of terminal deoxynucleotidyl transferase (TdT) (Boehringer-Mannheim) plus 30 μM fluoresceinated dUTP (Boehringer-Mannheim) in 1× TdT buffer for 1 h at 37°C. Cells were rinsed in PBS and visualized by epifluorescence using standard fluorescein excitation and emission filters. Statistical significance was assessed using Student's *t*-test.

### RESULTS

Injection studies using trypan blue established that up to 100 μl of reagent could be delivered into the medullary canal of the murine tibia without gross cortical fracture. Biodistribution studies in which radiolabelled MAD11 (2x10$^{6}$ c.p.m.) was injected into the medullary canal of the right tibia of three mice showed that, after 30 min, 30.001±3.381 c.p.m. of radioactivity was present in the injected bone. At the same time, the radioactivity detected in the contralateral legs was 340±148 c.p.m.

To determine whether the injection of MAD11 in tibial bone marrow might protect against doxorubicin-induced myelotoxicity-related death, 54 BALB/c mice were randomly divided into three

© Cancer Research Campaign 1997

**Figure 3** Effect of intratibial injection of MAD11 on doxorubicin-induced apoptosis. Bone marrow from (a) an untreated mouse, (b) a doxorubicin-treated mouse, (c and d) two doxorubicin-treated mice: (□) MAD11- treated right tibia (▣) and contralateral tibia. Values are the mean±s.d. of the percentage of apoptotic cells present in five fields. The treatment schedule was as described in Materials and methods

**Figure 4** Photomicrograph of bone marrow cells from MAD11-treated right tibia (**A**) and contralateral tibia (**B**) of a mouse treated with 16 mg $kg^{-1}$ body weight of doxorubicin. Apoptotic nuclei were visualized by labelling the fragmented DNA with fluoresceinated dUTP via the terminal transferase (TdT) reaction groups and treated with 300 µg of MAD11 in the right tibia, the same dose of the monoclonal antibody given i.v., or with 100 µl of saline intratibially, and after 10 min, all mice were treated i.p. with 14 mg $kg^{-1}$ body weight of doxorubicin. During the next 40 days, none of the mice in the intratibially MAD11-treated group died (0/19), whereas 33.3% (6/18) of mice treated i.v. and 41.2% (7/17) of mice treated only with doxorubicin died (Figure 1). The protection against mortality in intratibially MAD11-treated mice was statistically significant ($P<0.002$ *vs* doxorubicin only-treated mice; $P<0.008$ *vs* i.v. MAD11 plus doxorubicin-treated mice).

In a second experiment, 40 mice received a higher dose of doxorubicin (18 mg $kg^{-1}$ weight); 18 of these mice were pretreated intratibially with MAD11. In the first 6 days, no differences in the percentage of deaths were observed in the two groups [27.8% (5/18) in intratibially MAD11-treated mice and 27.3% (6/22) in mice treated with doxorubicin only]. In the surviving animals, no deaths were observed thereafter in intratibially MAD11-treated mice (0/13), whereas 50% (8/16) of mice treated with doxorubicin alone died ($P<0.003$).

To assess the effect of MAD11 that escaped into the bloodstream on the chemotherapeutic effect of doxorubicin, hybrid BD2-F1 mice were injected with P388 leukaemia cells and treated with doxorubicin alone or with doxorubicin plus 1000 µg of MAD11 in the right tibia. Death of all BD2-F1 mice, which tolerate high doses of doxorubicin, was caused by progression of the tumour; as shown in Figure 2, the survival curve of leukaemic BD2-F1 mice treated with doxorubicin and MAD11 was superimposable on that of mice receiving doxorubicin alone.

Doxorubicin has been reported to induce cytotoxic effects with the characteristics of apoptosis in cells from different tissues (Barry et al, 1990; Anilkumar et al, 1992), i.e. nuclear and cytoplasmic condensation and preservation of the organelles in the early stages. Fluorescence microscopy of bone marrow cells from mice injected i.p. with 16 mg $kg^{-1}$ body weight of doxorubicin revealed evidence of cell death in bone marrow obtained 2, 4, 8, 16 and 32 h after treatment. The morphological features were similar in all samples studied but considerable differences existed in the frequency of dead cells with a maximum at 8 h (unpublished results). To test whether intratibial injection of MAD11 protects bone marrow cells from doxorubicin-induced apoptosis, mice were injected in the right tibia with 50 µg of MAD11 and then treated i.p. with 16 mg $kg^{-1}$ body weight of doxorubicin. As shown in Figure 3 and 4, the percentage of apoptotic cells in the MAD11-treated tibia was significantly ($P<0.0001$, Figure 3c; $P<0.001$, Figure 3d) lower than in the contralateral tibia.

### DISCUSSION

In this study, we show that an anti-doxorubicin monoclonal antibody directly injected in the tibial bone of mice can protect the bone marrow against the toxic effects of high-dose doxorubicin.

Several growth factors and inhibitors that prevent chemotherapy-induced myelosuppression are now available. These reagents have a reversible stimulatory or inhibitory action on the cycling of early stem cells and precursors or late progenitors. In the past 10 years, much attention has focused on the rapid recovery induced by colony-stimulating factors (CSFs). However, the long-term effects should not be overlooked, and both overt and latent marrow failure represents a hazard (Tubiana et al, 1993). Moreover, some recent experimental data suggest that repeated growth factor stimulation may contribute to exhaustion of the stem cell pool (Hornung et al, 1992; Moore, 1992). Indeed, a network of finely tuned regulatory factors is required to control normal haemopoiesis or regeneration following an insult, and drugs can also affect haemopoietic stem cells indirectly by their toxicity to bone marrow stromal cells and damage to the stem cell microenvironment (Schofield, 1986). If the damage to haemopoietic stem cells is not ultimately lethal, depletion is followed by recovery at a rate that depends on the proliferation rate of colony-forming units

© Cancer Research Campaign 1997

(CFUs) and on the rate of differentiation into committed cell compartments (Vassort et al, 1971), both under the regulation of the stem cell microenvironment. Thus, new strategies to minimize damage to all bone marrow components should be explored.

Two different doses of doxorubicin were used in evaluating the protective effect of MAD11. With doxorubicin at 14 mg $kg^{-1}$ body weight, no death was observed in intratibially MAD11-treated mice during the entire experiment compared with a 41% death rate in mice treated with doxorubicin only. With 18 mg $kg^{-1}$ doxorubicin, a similar percentage of mortality was observed in the intratibially MAD11-treated groups and in control groups for the first 6 days; this early mortality can be ascribed to non-haematological reasons, since high doses of doxorubicin are also toxic for organs other than bone marrow, consistent with a previous study (Grzegorzewski et al, 1994) showing that bone marrow transplantation did not protect from acute doxorubicin toxicity. After the nadir of doxorubicin-induced myelodepression on day 7 (Mazué et al, 1995), statistically significant protection against mortality in intratibially MAD11-treated mice was observed.

The administration of the anti-doxorubicin monoclonal antibody before chemotherapeutic treatment was effective in reducing doxorubicin-induced apoptosis, raising the possibility that the increase in survival and the capacity to repopulate lethally irradiated mice is related to an inhibition of doxorubicin-induced apoptosis in bone marrow cells.

Future therapeutic strategies to prevent myelosuppression might involve the combination of inhibitory molecules able to maintain haematopoietic stem cells in a quiescent state temporarily, thus protecting them from intensive chemotherapy, together with cytokines, such as granulocyte colony-stimulating factor (G-CSF), which are capable of accelerating haematopoietic recovery. Other new strategies, such as antisense oligonucleotides, anti-receptor reagents and multidrug resistance gene therapy, also appear promising for clinical use.

Our data show that monoclonal antibodies directed against chemotherapeutic drugs can be used for the local protection of the bone marrow in mice receiving high-dose chemotherapy without interfering with the anti-tumour properties of the drug. Since children's long bones contain bone marrow and the cortical bone can be easily injected, the intratibial administration of this antibody could provide an alternative approach to protect bone marrow cells locally in young patients with solid tumours.

### ACKNOWLEDGEMENTS

This study was supported by the Associazione Italiana per la Ricerca sul Cancro and by Consiglio Nazionale Ricerche-progetto finalizzato Applicazioni Cliniche della Ricerca Oncologica. We thank E Randisi for technical collaboration, L Mameli for manuscript preparation and M Azzini for photographic reproductions.

### REFERENCES

Anilkumar TV, Sarraf CE, Hunt T and Alison MR (1992) The nature of cytotoxic drug – induced cell death in murine intestinal crypts. *Br J Cancer* **65**: 552–558

Balsari A, Morelli D, Ménard S, Tagliabue E, Colnaghi MI and Ghione M (1990) A new monoclonal antibody recognizing anthracyclinic molecule. *Anticancer Res* **10**: 129–132

Balsari A, Ménard S, Colnaghi MI and Ghione M (1991) Anti-drug monoclonal antibodies antagonize toxic effect more than anti-tumor activity of doxorubicin. *Int J Cancer* **47**: 889–892

Balsari AL, Morelli D, Ménard S, Veronesi U and Colnaghi MI (1994) Protection against doxorubicin-induced alopecia in rats by liposome-entrapped monoclonal antibodies. *FASEB J* **8**: 226–230

Barry MA, Behnke CA and Eastman A (1990) Activation of programmed cell death (apoptosis) by cisplatin, other anticancer drugs, toxins and hyperthermia. *Biochem Pharmacol* **40**: 2353–2362

Berlin Ö, Samid D, Donthineni-Rao R, Akeson W, Amiel D and Woods VL (1993) Development of a novel spontaneous metastasis model of human osteosarcoma transplanted orthotopically into bone of athymic mice. *Cancer Res* **53**: 4890–4895

Chabner BA and Myers CE (1993) Antitumor antibiotics. In *Cancer Principles and Practice of Oncology*, De Vita VT, Hellman S and Sosenberg SA (eds), pp. 374–384. Lippincott: Philadelphia.

Chesson BD, Lacerna L, Leyland-Jones B and Sarosy G RE (1989) Autologous bone marrow transplantation. Current status and future directions. *Ann Intern Med* **110**: 51–65

Dexter TM (1989) Regulation of hemopoietic cell growth and development: experimental and clinical studies. *Leukemia* **3**: 469–474

Eaves CJ, Cashman JD, Kay RJ, Dougherty GJ, Otsuka T, Gaboury LA, Hogge DE, Lansdorp PM, Eaves AC and Humphries RK (1991) Mechanisms that regulate the cell cycle status of very primitive hematopoietic cells in long-term human marrow cultures II. Analysis of positive and negative regulators produced by stromal cells within the adherent layer. *Blood* **78**: 110–117

Grzegorzewski K, Ruscetti FW, Usui N, Damia G, Longo DL, Carlino JA, Keller JR and Wiltrout RH (1994) Recombinant transforming growth factor beta1 and beta2 protect mice from acutely lethal doses of 5-fluorouracil and doxorubicin. *J Exp Med* **180**: 1047–1057

Guigon M, Lemoine F and Najman A (1994) Bone marrow protection. *Bone Marrow Transplant* **13**: 93–95

Gulati SC, Yahalom J and Portlock C (1991) Autologous bone marrow transplantation. *Curr Probl Cancer* **15**: 1–35

Hénon PR (1993) Peripheral blood stem cell transplantations: past present and future. *Stem Cells* **11**: 154–172

Hénon P, Liang H, Beck-Wirth G, Eisenmann JC, Lepers M, Wunder E and Kandel G (1992) Comparison of hematopoietic and immune recovery after autologous bone marrow or blood stem cell transplant. *Bone Marrow Transplant* **9**: 285–291

Hornung RL and Longo DL (1992) Hematopoietic stem cell depletion by restorative growth factor regimens during repeated high dose cyclophosphamide therapy. *Blood* **80**: 77–83

Lieschke GJ and Burgess AW (1992) Granulocyte stimulating factor and granulocyte–macrophage colony-stimulating factor. *N Engl J Med* **327**: 28–35

Mazué G, Iatropoulos M, Imondi A, Castellino S, Brughera M, Podesta A, Della Torre P and Moneta D (1995) Anthracyclines: a review of general and special toxicity studies. *Int J Oncol* **7**: 713–726

Metcalf D (1990) The colony stimulating factors discovery, development and clinical applications. *Cancer* **65**: 2185–2195

Moore MAS (1992) Does stem cell exhaustion result from combining hematopoietic growth factros with chemotherapy? If so, how do we prevent it. *Blood* **80**: 3–7

Morelli D, Ménard S, Colnaghi MI and Balsari A (1996) Oral administration of anti-doxorubicin monoclonal antibody prevents chemotherapy-induced gastrointestinal toxicity in mice. *Cancer Res* **56**: 2082–2085

Roberts MM, Haylock DN, Dyson PG, Branford AL, Thorp D, Ho JQK, Dart GW, Horvath N, Davy MLJ, Olweny CLM, Abdi E and Juttner CA (1992) Comparison of hematological recovery times and supportive care requirements of autologous recovery phase peripheral blood stem cell transplants, autologous bone marrow transplants and allogenic bone marrow transplants. *Bone Marrow Transplant* **9**: 277–284

Sardini A, Villa E, Morelli D, Ghione M, Ménard S, Colnaghi MI and Balsari A (1992) An anti-doxorubicin monoclonal antibody modulates kinetic and dynamic characteristics of the drug. *Int J Cancer* **50**: 617–620

Schofield R (1986) Assessment of cytotoxic injury to bone marrow. *Br J Cancer* **53**: 115–125

Tubiana M, Carde P and Friendel E (1993) Ways of minimising hematopoietic damage induced by radiation and cytostatic drugs – the possible role of inhibitors. *Radiother Oncol* **29**: 1–17

Vassort F, Frindel E and Tubiana M (1971) Effects of hydroxyurea on the kinetics of colony forming units of bone marrow in the mouse. *Cell Tissue Kinet* **4**: 423–431

Weiss RB (1992) The anthracyclines: will we ever find a better doxorubicin? *Semin Oncol* **19**: 670–686

© Cancer Research Campaign 1997